# A Report on Fungal (1→3)-α-d-glucans: Properties, Functions and Application

**DOI:** 10.3390/molecules24213972

**Published:** 2019-11-02

**Authors:** Katarzyna Złotko, Adrian Wiater, Adam Waśko, Małgorzata Pleszczyńska, Roman Paduch, Jolanta Jaroszuk-Ściseł, Andrzej Bieganowski

**Affiliations:** 1 Institute of Agrophysics, Polish Academy of Sciences, Doświadczalna 4, 20-290 Lublin, Poland; a.bieganowski@ipan.lublin.pl; 2 Department of Industrial and Environmental Microbiology, Maria Curie-Skłodowska University, Akademicka 19, 20-033 Lublin, Poland; adrianw2@poczta.umcs.lublin.pl (A.W.); m.pleszczynska@poczta.umcs.lublin.pl (M.P.); jolanta.jaroszuk-scisel@poczta.umcs.lublin.pl (J.J.-Ś.); 3 Department of Biotechnology, Human Nutrition and Food Commodity Science, University of Life Sciences in Lublin, Skromna 8, 20-704 Lublin, Poland; awasko1@tlen.pl; 4 Department of Virology and Immunology, Maria Curie-Skłodowska University, ul. Akademicka 19, 20-033 Lublin, Poland; rpaduch@poczta.umcs.lublin.pl; 5 Department of General Ophthalmology, Medical University, Chmielna 1, 20-079 Lublin, Poland

**Keywords:** fungi, cell walls, polysaccharides, (1→3)-α-d-glucans

## Abstract

The cell walls of fungi are composed of glycoproteins, chitin, and α- and β-glucans. Although there are many reports on β-glucans, α-glucan polysaccharides are not yet fully understood. This review characterizes the physicochemical properties and functions of (1→3)-α-d-glucans. Particular attention has been paid to practical application and the effect of glucans in various respects, taking into account unfavourable effects and potential use. The role of α-glucans in plant infection has been proven, and collected facts have confirmed the characteristics of *Aspergillus fumigatus* infection associated with the presence of glucan in fungal cell wall. Like β-glucans, there are now evidence that α-glucans can also stimulate the immune system. Moreover, α-d-glucans have the ability to induce mutanases and can thus decompose plaque.

## 1. Introduction

Glucans are glucose polymers and are, therefore, classified as polysaccharides. Although their simple composition might be taken as indicative of an uncomplicated construction, differences can be found among glucans in terms of the anomeric configuration of glucose units and glycosidic linkages (sequence and position), the type and degree of bond branching and molecular size [1]. Glucans can, therefore, be divided according to the anomeric conformation of glucose (α-glucans, β-glucans and α,β-glucans) as well as the position of the glycosidic bonds (1,3-, 1,4- and 1,6-glucans) [2].

**Figure 1:**
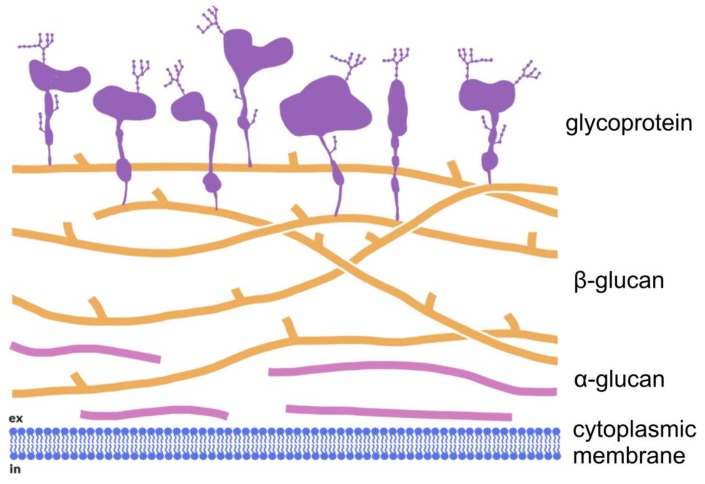
Structure of fungal cell walls by Grün [3].

Along with glycoproteins, glucans are components of fungal cell walls. These compounds shape fungal cells and form a barrier, which protects the cells against environmental stress. The outermost layer of the cell wall consists of glycoproteins, and beneath this is an inner layer, which is formed by glucose polymers (β-1,3-glucans, β-1,6-glucans, α-1,3-glucans and chitin) [3,4]. It has been proved that β-glucans are connected by covalent bonds with chitin chains, and this network is under the glycoprotein layer. There are no unambiguous reports as to the position of α-glucans chains. Grün reported that the (1→3)-α-d-glucans layer is beneath all the layers of biopolymers, just above the cytoplasmic membrane (Figure 1) [3]. However, the latest reports indicate that the location of (1→3)-α-d-glucans is quite fluid and depends on many factors, including the fungus species [5,6]. In addition, within the same species (*A. fumigatus*), (1→3)-α-d-glucans may be in a different location, depending on the developmental form of the fungus (conidia or vegetative mycelium) [5] and the type of plant cultures [6].

The fluorescently labelled antibodies method is used to determine the location of α α-glucans in cells [7,8]. Choma et al. (2013) found (1→3)-α-d-glucan in the hyphae of *Aspergillus wentii* and particularly large quantities in hyphal septa [7]. Fujikawa, Kuga and Yano et al. (2009) used fluorescent labels to study the distribution of infected cell wall polysaccharides in the rice blast fungus *Magnaporthe grisea*. The results obtained with immunoelectron microscopy showed that α-1,3-glucan and β-1,3-glucan were mixed in the cell wall of hyphae, but a larger deployment of α-1,3-glucan occurred further from the cell membrane [8].

(1→3)-α-d-glucans have been found in numerous fungi [8,9,10,11]. In larger quantities, α-glucans occur in representatives of the *Basidiomycetes* class. Their content reaches 44–53% of the dry weight of fruiting bodies of the birch pathogen *Fomitopsis betulina* (Bull.: Fr.), and even 75–88% in the fruiting bodies of *Laetiporus sulphureus* (Bull.: Fr.) Murrill [12,13]. In smaller quantities, α-glucans were found in representatives of the *Ascomycetes* class, i.e., *Aspergillus niger* (9%). Some kinds of yeast may not have α-glucans (e.g., *Saccharomyces cerevisiae* and *Candida albicans*) [14], but *Histoplasma capsulantum* contains 46.5% [15]. Table 1 presents an overview of the latest literature (since 2000) relating to the content of (1→3)-α-d-glucans and their properties in individual species of fungi [7,16,17,18,19,20,21,22,23,24,25]. The table is a supplement to the information contained in Grün’s publication, which provided an analogous review of the literature on (1→3)-α-d-glucans content in individual fungi [3]. Thus, both works fully document the occurrence of (1→3)-α-d-glucans in fungi.

Biosynthesis of α-glucans has not been fully researched. In general, the synthesis of these polysaccharides involves glucose and (1→3)-α-d-glucan synthase [26]. Grün (2003) indicated two (1→3)-α-d-glucans biosynthesis mechanisms [3]. The first is a single-step biosynthesis mechanism, which occurs in the walls of the spores and involves a single α-glucan monomer. The two-step mechanism in *Schizosaccharomyces pombe* involves α-glucan, which has a dimeric structure composed of two covalently linked building blocks, each consisting of a linear (1→3)-α-d-glucan segment with a small number of (1→4)-residues at its reducing end. In another publication, Grün et al. studied biosynthesis of α-glucans in yeast [27]. Hochstenbach et al. (1998) specifically identified α-glucan synthase in *Schizosaccharomyces pombe*, i.e., Ags1p and its three domains: an intracellular synthase domain, a C-terminal multipass transmembrane domain, and an N-terminal extracellular domain, which might act as a transglycosylase [28]. *Aspergillus nidulans* has two α-1,3-glucan synthase genes (agsA and agsB), but studies show that only AgsB is necessary for normal growth [29]. Yoshimi et al. (2017) have extensively described the α-glucan biosynthesis mechanism, including issues related to signalling the integrity of the cell wall, the genes and enzymes involved in this process, and a detailed description of the biosynthesis in *Schizosaccharomyces pombe* [30]. They have also suggested a biosynthesis and degradation model for (1→3)-α-d-glucan in *Aspergillus nidulans.*

In the literature, there is a lot of information about fungal β-glucans, but α-glucans (which are also frequently occurring polysaccharides in fungi) have not yet been fully characterized. The starting point for discussion is the work of Grün from 2003 [3]. In recent years, a variety of analyses of α-glucans have been performed, shedding light on the structure, properties and functions of these polymers. However, a conclusive report on cell wall location and the functions of α-glucans is missing. For this reason, it is worthwhile collecting information about these as yet little-known polysaccharides. The report presented here is one of the few reviews on the subject of α-glucans. In addition to current information on their structure and functions, the review describes miscellaneous applications of (1→3)-α-d-glucans, e.g., as a mutanase inducer [31,32,33,34,35], as support for lipase immobilization, as a prebiotic [36] and, finally, based on recent research undertaken by our group, a new application as a heavy metal sorbent [37,38].

## 2. Methods for Isolating (1→3)-α-d-Glucans

In the literature, there are several procedures for isolating α-glucans [4]. Table 2 shows the different isolation methods and compares the reagents used in these. Generally, the methods presented consist of the decolourization stage, which is also the stage of removing the water-soluble fraction, further obtaining the alkali-soluble fraction, neutralization and rinsing [39,40,41,42].

For α-glucans isolation, Grün [3] used different reagents and a method of isolating. The milled fruiting bodies of *Laetiporus sulphureus* were treated with a solution containing EDTA, Tris and pH 7.6 solution, and these were then blended. SDS and 2-mercaptoethanol were added and boiled to remove cytosolic impurities. The suspension was centrifuged and washed in water. Following this, the milling and extraction steps were repeated. The centrifuged material was suspended in ice-cold NaBH_4_ and KOH, and stirred for 30 min at 4 °C. The undissolved material was removed by centrifugation. Acetic acid was added to the centrifuged supernatant to bring the solution to pH 6.0. The water-insoluble fraction was mixed at 4 °C for 24 h, after which the pellet was centrifuged and resuspended in sodium azide, 2-mercaptoethanol, citrate-phosphate buffer and pH 5.3 containing Zymolyase-100T (Seikagaku, Tokyo, Japan) to remove (1→3)-β-d-glucans. After stirring overnight at 37 °C, the insoluble fraction was collected by centrifugation and washed twice with water. Alkaline extraction and enzymatic hydrolysis were repeated once more. Finally, the material was re-extracted with SDS and 2-mercaptoethanol and washed with sodium azide.

## 3. Structure and Property of Fungal (1→3)-α-d-Glucans

The properties of compounds (especially biologically active molecules) depend on their structure, conformation and molecular weight. In order to determine the structure, molecular weight and other physicochemical properties of (1→3)-α-d-glucans, different techniques are used: spectroscopic, chemical, and separation methods [1], for example, size-exclusion (gel permeation) chromatography (SEC or GPC), laser light scattering (LLS), and viscometry. This analysis is difficult due to α-glucans being insoluble in water, so specific solvents or chemical modification are required [1].

(1→3)-α-d-glucans consist of glucose monomers linked with mainly 1,3-glycosidic bonds, but their structure varies depending on the fungus species. However, in α-glucans, there are not only 1,3 bonds (such as those in the *Amanita muscaria*) [40], but there may also be small amounts of 1,4- linkages (*Neurospora crassa*) [43] as well as compounds of the nigeran type, where alternate 1,3- and 1,4- bonds exist (*Parmelia caperata*) [44]. The structure of alkali-soluble polysaccharides isolated from *Boletus edulis* was also studied. This polymer consists mainly of α-(1→3)-d-glucan chains (about 67%) but also α-(1→3)-d-mannans (28%) [38]. In another construction, α-glucans form on dental plaque, where the glucose units are linked by binding 1,3- in the main chain and 1,6- in the side chains [26].

(1→3)-α-d-glucans are insoluble in water due to the presence of strong hydrogen bonds. The presence of these bonds makes it difficult to determine the molecular weight or conformation of the molecules. Zhang et al. (2000) [21] used urea and NaOH solutions to break the hydrogen bonds, and determined the molecular weight and viscosity of the α-glucan from the *Lentinus edodes* as 5.21 × 10^5^ and 148 cm^3^·g^−1^, respectively. The molecular weight of the glucan was directly influenced by the degree of polymerization, which was between 60 and 3500 [3].

**Figure 2:**
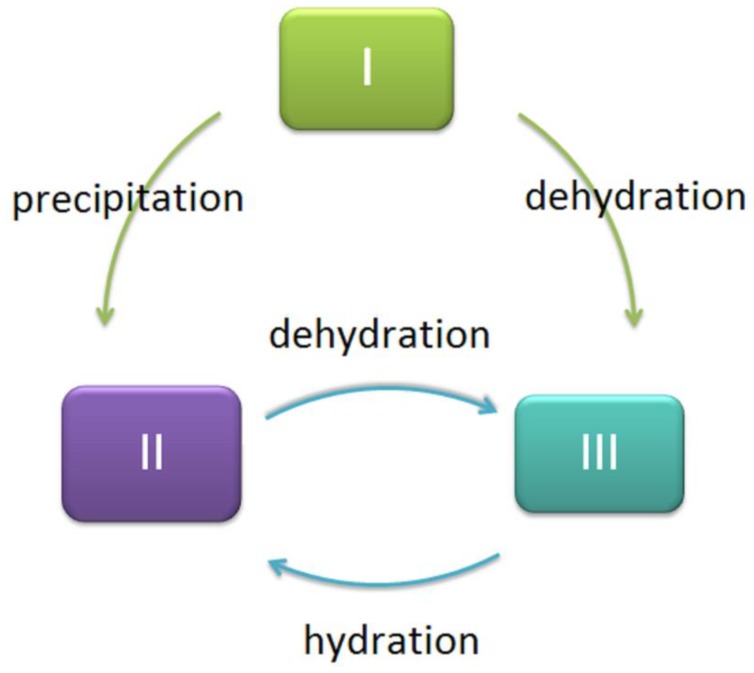
Mutual transformations of crystalline/polymorphic forms of (1→3)-α-d-glucans, based on Jelsma et al. [13].

By 1979, the structure of (1→3)-α-d-glucans had already been defined by Ogawa et al. [45], who used X-ray analysis and defined conformation as extended, close to a 2/1 helix [45,46]. Moreover, in LiCl/DMSO solution, (1→3)-α-d-glucans occurs as a flexible chain [47,48] and (according to other reports) as a random coil [20] due to the breaking of hydrogen bonds. 

(1→3)-α-d-glucans occur in different crystal forms (polymorphs), depending on the fungus species, type of tissue investigated (reproductive or non-reproductive tissue) and preparation conditions [13]. Jelsma and Kreger (1979) [13] investigated four species of fungi using X-ray diffraction: *Laetiporus sulphureus, Piptoporus betulinus, Schizophyllum commune*, and *Aspergillus nidulans*. They reported that α-glucan can present in three polymorphs (I, II and III). The first form (I) occurs in native tissue of *L. sulphureus* and *P. betulinus*. The second form (II) can be obtained by precipitation from alkaline solution. The third crystalline form is not hydrated, in contrast to the other two, and can, therefore, be obtained by drying polymorph I at 60 °C (total change) and polymorph II at 95 °C in a vacuum. It seems that form II is the most energy stable, but the less stable form is generally preferred in nature [13]. These changes are presented schematically in Figure 2, based on Jelsm’s work [13].

Choma et al. (2013) [7] characterized water-insoluble polysaccharide from *Aspergillus wentii* and reported that the studied polymer was linear α-d-glucan with a molecular weight of 850 kDa. The glucan was composed of 25 subunits, and each subunit consisted of 200 α-d-glucose residuals with (1→3)-bonds separated by short fragments of α-d-glucoses linked by (1→4)-bonds.

## 4. Functions of (1→3)-α-d-Glucans

The role of cell wall components of fungi is generally known; they provide cell rigidity to protect against the external environment and, at the same time, provide flexibility, which enables cell growth. The typical role of α-glucans in cell walls is controversial [3]. Zonneveld (1972) reported that α-glucan in *Aspergillus nidulans* stores material which has accumulated in the cell wall during vegetation growth [49]. However, the mutant of this fungus which lacked α-glucan showed normal growth, suggesting that α-glucan in *Aspergillus nidulans* is an unnecessary compound for this process [50]. Glucans play a significant role in yeast because they are essential for ensuring the integrity of fission yeast cells and are a major constituent of fission yeast spore walls. The spore wall (like the cell wall of haploid cells) maintains structural integrity, regulates cell permeability and protects the cell from environmental stresses [3]. Moreover, the observed correlation between a lack of α-glucans in the cell wall of fungus is temperature sensitive. The mutant of *Schizosaccharomyces pombe* (with a mutation in the gene responsible for α-glucan biosynthesis) has been found to be sensitive to temperature and its cells wall lysed at 37 °C [28].

In a medical context, extensive interest in glucans is associated with their anti-tumour properties. More specifically, β-glucans have these properties, but α-glucans have little or no anti-tumour effect due to being insoluble in water. However, modified α-glucans (for example, carboxymethylated or sulphated derivatives) are soluble in water and have potent anti-tumour activity [22,51,52,53]. Wiater et al. (2011) reported that carboxymethylated α-glucans from *Lentinus edodes, Pleurotus ostreatus, Piptoporus betulinus* and *Laetiporus sulphurous* have a biological activity potential (express cytotoxic or mitochondrial metabolism-modulating effects), but they do not show free radical scavenging activity [22]. Moreover, glucan-based preparations that stimulate the immune system are commercially available. Bao et al. (2001) reported that modified α-glucans (carboxymethylated derivatives) have stimulating effects on lymphocyte proliferation and antibody production [54].

In addition, the report by Yoshimi et al. [30] cited diverse applications of glucans, e.g., a possible use as thermoplastic materials due to their thermostable properties [55] or in the fermentation industry due to enzymes and metabolites secreted by fungi [56].

The presence of α-glucans also has a negative effect, and the correlation between the presence of α-glucan and fungal virulence is known [57,58]. Fujikawa et al. [8,59] report that many fungi that cause plant diseases through the presence of α-glucans impair the immune response of plants, and, in some species, these polysaccharides are used to maintain the infectivity of structures.

Moreover, α-glucans enables aggregation of swollen conidia, which occurs in biofilm during pulmonary aspergilloma (caused by *A. fumigatus*) [60]. It has been proven that α-glucan in mutans streptococci causes virulence in the aetiology of dental caries in humans through the glucans’ cell–cell and cell–surface adhesion, especially adhesion to hard surfaces [61,62,63]. On the other hand, by using α-glucans for the production of (1→3)-α-glucanases, glucosidic bonds in the mutant synthesized by cariogenic oral streptococci can be hydrolysed, thereby removing dental plaque mutans [64,65,66].

Table 3 provides a summary of the medical properties of fungal (1→3)-α-d-glucans [16,22,25,33,34,36,42,51,52,53,54,57,58,60,64,65,66,67,68,69,70,71,72,73,74,75,76,77,78,79,80,81,82,83,84].

## 5. The Biological Role of (1→3)-α-d-GLucans

### 5.1. Fungal (1→3)-α-d-Glucans Is Essential for Successful Plants Infection

The effect of fungus–plant host interaction results from the establishment of a balance between fungal virulence and plant resistance [85,86,87,88]. Disturbance of the balance (resulting from a lowered plant self-protection ability or increased fungal virulence) leads to development of disease [89,90,91,92]. The fungus–plant interaction constitutes a dynamic system involving numerous processes directly inhibiting pathogen growth and indirectly affecting the growth of both organisms (resistance induction) as well as co-regulation of metabolite formation [93]. Plant protection against pathogen invasion (provided by the direct action of microorganisms) is enhanced by indirect induction of plant resistance by elicitors produced during inhibition of pathogen growth by rhizosphere microorganisms employing mechanisms of direct action [94,95,96].

Cell walls are the most important sources of molecular designs associative with microorganisms/pathogens (microbe/pathogen-associated molecular patterns, MAMPs/PAMPs) [8,10]. Fungal MAMPs include β-glucans, mannans and chitin, which induce an immune response in both plant and mammalian cells [11,46,97,98,99]. They are identified by binding proteins that make up the receptor complex. Chitin and β-1,3-glucan in fungal walls are the most important and best known MAMPs, which cause an immune response in plants [11,100,101,102].

Elicitors are plant-resistance inducers released from the cell walls of a host (endoelicitors) and invading microorganism (exoelicitors). Elicitors already recognized can be classified as members of the race-specific group that determines induction of gene-to-gene resistance. They can also be included in the particularly interesting group of common elicitors of so-called race non-specific resistance, which interact with all varieties of an infected host species and originate from various pathotypes of pathogenic and/or non-pathogenic strains [85,103,104,105,106,107].

The latest reports indicate that (1→3)-α-d-glucans may be a factor in the virulence of some pathogens [50], with a role in the aggregation of germinating conidia of *Aspergillus nidulans* [108] and showing elicitor activity [9,109]. Compared with β-glucans, α-glucans are less well understood in terms of their ability to induce plant resistance and their anti-cancer properties. Water-soluble α-glucan from the lichen fungus *Ramalina celastri* (a linear polymer of glucose with α-(1,3)- and α-(1,4)- in a ratio of 3:1) has an in vitro anti-cancer property against HeLa cells and in vivo murine macrophage-macrophage activation capacity, stimulating phagocytosis and increasing the production of hydrogen peroxide [67]. (1→3)-α-d-glucan presents in the wall of the non-pathogenic binucleated fungus *Rhizoctonia* (BNR) (an effective factor in biological protection, inducing plant resistance against diseases caused by pathogenic strains of *R. solani*) has been found to not only induce resistance to the infection in potato stems but also induce protection against cancer and dry rot caused by *Rhizoctonia* [110,111,112]. The (1→3)-α-d-glucans strongly induce activity markers of resistance-related proteins of pathogenicity (PR): (1→3)-β-glucanase (PR-2) and chitinase (PR3) [113]. Kinetics of induction of (1→3)-β-glucanase in the stems of potato mycelium are similar to those induced by (1→3)-α-d-glucan isolated from the cell wall of BNR. Wolski et al. [112] have, for the first time, detected α-glucanase activity in plant tissues using (1→3)-α-d-glucans as a substrate (derived from a *Rhizoctonia* cell wall). It has been shown that accumulation of (1→3)-α-d-glucan depends on the MAP kinase pathway (Mps1), which can be activated by an endoelicitor, such as that originating from degradation of wax 1.16-hexadekanodiol [8]. Rappleye et al. (2007) proved that the (1→3)-α-d-glucan both spatially and functionally masks the (1→3)-β-d-glucan in infection hyphae of *Histoplasma capsulatum* (and protects them against enzymatic degradation) by blocking the plant host’s ability to recognize these polymers [10].

It seems that the key function of α-glucans are protection of β-glucans and chitin against degrading enzymes (that is, against the release of exoelicitors), against recognition by the plant and for running the defence response [8,10,114]. This mechanism seems to be universal and can be observed in both fungal-mammal [10] and fungal–plant interaction [8,30]. There seems to be a clear relationship between the amount of α-glucan in fungal cell wall (FCW) and the ability of a fungus to infect host cells (virulence). Studies contributing to our understanding of the fungal-host defence response prevention strategy have indicated the location of polysaccharides in cell walls using fluorescent marking during differentiation of infection structures in *Magnaporthe grisea* [8,115]. Immunomicroscopic analysis showed that infectious cell wall fragments of rice pathogen *Magnaporthe grisea* (1→3)-α-d-glucan and (1→3)-β-d-glucan are intertwined with each other, but (1→3)-α-d-glucan remains further away from the cell membrane. When infective *Magnaporthe grisea* hypha was developing on a hydrophobic plastic surface, (1→3)-α-d-glucan was only found in an appressorium. On the other hand, when infective *Magnaporthe grisea* hypha developed on the surface of plants, (1→3)-α-d-glucan was found with both hypha-growing from a conidium (germ tube) and an appressorium [115].

**Figure 3:**
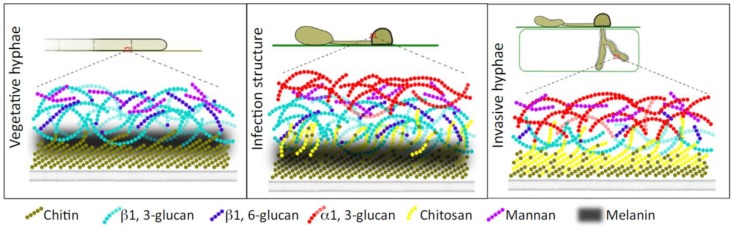
Changes in the structure of the fungus cell wall during plant infection, according to Geoghegan et al. [117].

Fungal plant pathogens reorganize their cell wall components in response to specific plant- derived compounds, which these pathogens may encounter during infection [116]. Figure 3 shows specific changes in the fungal cell wall structure during plant infection in a simplified manner as the exact distribution of polysaccharides in the wall is unknown. In addition, the arrangement of cell wall proteins has been omitted for the sake of clarity. Chitin, (1→3)-β- and (1→6)-β-glucans are present in all cell types and form a specific grid. The extracellular matrix (EM) is the glycoprotein outer layer. Plant infection causes deacetylation of chitin, forming chitosan and causing a decrease in β-glucans content. The distribution of (1→3)-α-d-glucans also changes, occupying the outer layers [117].

Because (1→3)-α-d-glucans are a refractory polysaccharides for plants, it is highly likely that surface accumulation of (1→3)-α-d-glucans protects the fungal cell walls from antifungal agents, such as cell wall degrading enzymes, which fungi encounter during infection [8].

### 5.2. The Role of (1→3)-α-d-Glucans in the Pathogenicity of Aspergillus Fumigatus

*Aspergillus fumigatus* is a human pathogen that causes systemic infections in immunodeficient patients, for example, after transplantation or chemotherapy. Ghazaei et al. prepared a review on pathogenesis and infection by *Aspergillus fumigatus*, where they discussed various factors affecting the virulence of this fungus (e.g., thermotolerance, nutrient uptake, adhesins, toxins) including the effect of cell wall components [80]. The cell wall of *Aspergilus fumigatus* is mainly composed of (1→3)-α-d-glucan [79]. This polysaccharide is present in the extracellular matrix which surrounds the fibrillar core, composed of (1→3)-β-d-glucan and chitin [58]. In *A. fumigatus*, α-glucan has a structural role in the cell wall, providing cell rigidity. The absence of this polymer in cells is compensated for by increasing chitin and/or β-glucan content, so it can be concluded that α-glucan is a key component of the cell wall and is not required for vegetative growth [118]. However, α-glucan participates in the process of germination of *Aspergillus fumigatus* conidia, more precisely, in the aggregation of germinating conidia. This aggregation (i.e., cell-cell interaction) is characteristic of the *Phanerochaete, Syncephalastrum,* and *Aspergillus* species [119,120,121,122,123]. An experiment conducted by Fontaine and Beauvais (2010) using beads coated with (1→3)-α-d-glucan has shown that these polysaccharide chains interact with one another, and the results confirmed the ability of α-glucan to aggregate conidia [60]. On the other hand, Henry et al. (2011) reported that a lack of α-glucan does not disrupt the process of conidial germination. The germination level of the mutant (without genes determining the biosynthesis of α-glucan) was found to be similar to the parental strain [118].

In *A. fumigatus*, α-glucan also has a different role, that is, this polysaccharide causes virulence. Removal of the gene responsible for its biosynthesis does not always limit its adverse effects. Biosynthesis of α-glucan is determined by three genes successively known as *AGS1*, *AGS2,* and *AGS3* [58]. Decreased α-glucan content in the cell wall only causes a lack of the gene *AGS1*. However, even the loss of α-glucan is enough to keep the virulence of *A. fumigatus*, and deletion of *AGS1* and *AGS2* does not reduce virulence in the strain [57,79]. There are reports that *AGS3* has a role in the biosynthesis of α-glucan and changes the virulence of *A. fumigatus* [79]. In an experiment by Maubon and Spark (2006), a strain lacking this gene was shown to produce faster and more robust disease compared with the original strain [79]. Its virulence was related to greater resistance to H_2_O_2_, the ability to germinate more rapidly [81] and an increase in melanin in the cell wall of conidia (which results in a resistance to phagocytic killing) [82]. According to Beauvais, Bozza, and Kniemeyer (2013) [58], deletion of the three genes results in a lack of (1→3)-α-d-glucans in fungal wall cells but no reduction in growth of the fungi. These mutant fungi are less pathogenic than the strain without modification. Deletion changes the structure of conidial cell walls, causing a reduction in the viability of conidia in vivo, that is, a decrease in the virulence of the mutant. A study using a model of murine aspergillosi reported that a mutant without α-glucan was less virulent than the native strain [58]. A lack of germination and vegetative growth was found in the lungs of mice infected with mutated conidia. Structural changes in the cell wall of mutant conidia caused conidia to be more easily phagocytosed by macrophages. In addition, unlike the native strain, β-glucan and chitin could be seen on the surface of mutant germinating conidia. These polysaccharides cause an immune response against fungi [6].

Beauvais et al. (2005) [57] suggest that α-glucan in the cell wall of *A. fumigatus* has an additional function to other pathogenic fungi. In *A. fumigatus*, α-glucan acts as a cement for the other cell wall components. It does not cause masking or anchoring of the cell wall surface molecules, which is probably what causes pathogenicity. For this reason, there are differences in the virulence of different species of pathogenic fungi; absence of the α-glucan synthetase gene does not reduce the virulence of the mutant when compared to the native strain [57].

Komarova et al. have used the relationship between the presence of (1→3)-α-d-glucan and the virulence in *Aspergillus fumigatus* in practice. They synthesized a pentasaccharide and neoglycoconjugates, which are related to α-glucan. This discovery will be used in the future to develop a diagnostic test system and vaccine to detect and combat this pathogen [83].

## 6. Applications of (1→3)-α-d-Glucans

### 6.1. Immunological Activity

Polysaccharides have been found to be good modulators and stimulators of immune responses. The most active include not only (1→3)-, (1→4)- and (1→6)-β-d-glucans but also (1→4)-, (1→6)- and (1→3)-α-d-glucans. These are considered as Toll-like receptor 4 (TLR4) ligands, and they induce receptor-mediated signal transduction via TLR4/IKK/NK-κB molecular pathways. Polysaccharides, including glucans, are known to induce activity and differentiation of immune cells, such as macrophages, leukocytes, or natural killer (NK) cells. These compounds belong both to innate (mainly macrophage-dependent immune system responses) and adaptive immunity (including B- and T-cell activity). Among them, monocyte activation is considered to be an initiating stage of immune responses after glucan impact [124]. Further immunomodulatory activity includes other cells like subsets of lymphocytes, NK, dendritic and myeloid cells, and their soluble mediators. (1→4)-α-d-glucans induce TNF-α, nitric oxide (NO) and prostaglandin E2 (PGE2) production by macrophages, as well as pro- and anti-inflammatory cytokines (such as IL-1β, IL-6, IL-8, IL-12, IL-18 or IFN-γ) by lymphocytes and other immunocompetent cells [125,126].

For a considerable time, (1→3)-α-d-glucan molecules were thought to have no significant biological activity. Nevertheless, given the source of the glucan, chemical modification of its structure and the presence of specific receptors on immune cells (which recognize glucan epitopes), it can exert a significant immunomodulatory effect. Currently, α-d-glucans are considered as promising compounds useful as adjuvants, especially for mucosal vaccination [126]. This activity is mediated through specific processes, including upregulation of MHC and co-stimulatory molecules, enhancement of antigen presentation, complement pathways and secretion of soluble mediators, as well as activation and stimulation of phagocytic cell motility [126]. Despite the multifaceted activity of α-d-glucans on immune responses, their immunomodulatory activity can be targeted through changes in the polymer length, molecular weight, branching degree and type of bonds, as well as solubility and charge of the molecule [126]. It has been shown that α-d-glucans with a mass higher than 10 kDa express poorer macrophage lineage activation properties than polymers with lower molecular mass [74,126]. This is clearly not always the case, however, and other variants often appear demonstrating the reverse. As with many other antigens, α-d-glucan charge is also important in the stimulation of immune cells, indicating that molecules with a high charge are often poorly immunogenic. Similarly, immune response is dependent on the type of glycosidic bonds, indicating differences in the stimulation of macrophage activity dependent on prevalence of (1→3)-α- or (1→6)-α- bonds within the polymer. Nonetheless, soluble and insoluble (1→3)-α-d-glucans or (1→6)-α-d-glucans are able to stimulate an immune response and soluble mediator secretion by immunocompetent cells. However, water-soluble forms are postulated to be more immunogenic. Modification of α-d-glucans like sulphation, aminopropylation, hydroxyethylation, or methylation also causes these agents to become much more biologically active than the native, underivatized components. Moreover, after chemical modification of glucans, they express less systemic toxicity than approved cytostatics [16,19,68,127]. These modifications also make polymers more soluble in water and, finally, may increase their biological activity. Chemical modification of α-d-glucans is thus an important procedure leading to an increase in the solubility of glucans in water, with simultaneous enhancement of their immunomodulatory activity [25].

Immune cells may be directly affected by α-d-glucans, mainly through binding to specific TLRs, CRs or dectin-1/2, expressed on the surface of these cells. This activation is followed by, e.g., NF-κB molecular pathway activation, leading to production of pro- and anti-inflammatory cytokines. These mediators stimulate antigen presentation in dendritic cells (DCs) and phagocytosis or secretion of other cytokines macrophages. The appropriate micro-environment, conditioned by the presence of glucan, also determines the most effective stimulation of the immune system and thus effective defence of the body against pathogens or neoplastic transformed cells [75]. However, the immunomodulatory and anti-tumour activities of α-d-glucans are generally mediated via host immune system activation rather than direct cytotoxic impact on target cells [123]. Stephen-Victor et al. reported that α-(1,3)-glucan isolated from *A. fumigatus* may play an important role in vaccination settings in patients after infections with this fungal pathogen [73]. The anti-tumour activity of glucans is mainly mediated by T lymphocyte activity and thymus-dependent mechanisms [69]. Maitake α-D-glucan (YM-2A; *Grifola frondosa*) has been found to enhance host anti-tumour action, activating CD4+ and CD8+ lymphocytes in the spleen and CD8+ cells in tumour-draining lymph nodes. This compound increases anti-tumour activity in CD11b+ myeloid cells, macrophages of the peritoneum and DCs. Moreover, this polymer stimulates secretion of soluble mediators in Peyer’s patches, indicating systemic activation of an anti-tumour immune response in the host [70]. Nevertheless, one cannot exclude direct anti-cancer or cytotoxic activity of α-d-glucans inducing apoptosis of transformed cells [67].

The role of these compounds is based on general tumourigenesis prevention. In particular, it should include limitation of tumour cell proliferation (especially sulphated forms of glucans), potentiation of the immune response against pathologic tissue, as well as preventive effects on the motility and metastasis of cancer cells. Moreover, they may induce tumour mass infiltration by natural killer (NK) cells and cytotoxic T lymphocytes, and consequently limit the volume of tumour growth [71]. Potential α-d-glucans anti-tumour activity should thus be linked (besides immune) with nervous or hormone systems stimulation and consequently whole-body mobilization [69,72,128].

### 6.2. (1→3)-α-d-Glucans as a Mutanase Inducers

(1→3)-α-d-glucans isolated from fungal cell walls can be also used for the production of (1→3)-α-glucanases, which are inducible enzymes catalysing the hydrolysis of glucosidic bonds in various (1→3)-α-d-glucans. (1→3)-α-glucanases are produced by microorganisms, fungi and bacteria, whose enzymes are involved in the processes of nutrition, including, inter alia, mobilization of cell wall α-d-glucans in response to carbon and energy source exhaustion [49,129], or, in the case of some yeasts, participate in morphogenetic processes during development and differentiation [130,131]. Some (1→3)-α-glucanases (also called mutanases) have the ability to hydrolyse the glucosidic bonds in mutans (branch (1→3)- and (1→6)-α-d-glucans) synthesized by cariogenic oral streptococci. Mutans are a key component of extracellular polymeric substances, forming a framework of cariogenic biofilm (dental plaque) [64,65,66]. When mutanases not naturally present in the oral cavity are added to oral hygiene products (such as mouth rinses, toothpaste or chewing gum), they can effectively support the cleaning of teeth through decomposition of dental plaque mutans [76]. Unfortunately, production of microbial mutanases is hampered by the lack of an available inducer for the synthesis of these enzymes. Mutan would be the best inducer if it were not for the potential pathogenicity of its producers, low synthesis yield and great structural diversity. However, it is possible to replace mutan in this role with more easily available fungal (1→3)-α-d-glucans. Cell walls preparations of various fungi or glucans with different levels of purity have been used for this purpose. High glucanase activities were obtained from *Bacillus circulans* WL-12 by supplementing the media with whole *Schizosaccharomyces pombe* cells or purified linear (1→3)-α-d-glucan (pseudonigeran) from *Aspergillus niger* [77]. Pseudonigeran was also used to induce *Trichoderma viride* glucanase [78]. Imai et al. (1977) [132] produced mutanase by culturing *Streptomyces* KI-8 on (1→3)-α-d-glucan isolated from dried fruiting bodies of *Lentinus edodes*. A cell wall preparation of *Schizophyllum commune* or *Botritis cinerea* was used as a (1→3)-α-glucanase inducer in *Bacillus circulans* KA-304 [31,32] and in *Trichoderma asperellum*, respectively [33]. The fruiting bodies of *Laetiporus sulphureus* are a particularly good source of the inducer for fungal and bacterial mutanases [34,42]. This basidiomycetous fungus grows worldwide on dead or weakened trees, producing very large, edible fruiting bodies and can be readily cultivated on a large scale [35]. A relatively high amount of (1→3)-α-d-glucan in the cell wall material of the fungus (up to 80%, which is several times more than other fungi) [3] can make the production of mutanases profitable.

### 6.3. (1→3)-α-d-Glucans as a Prebiotic

Intestinal microflora affects overall health, including the risk of cancer, so it is important for humans to maintain a balance of intestinal flora. For this reason, food and new probiotics are sought, which have a positive effect on intestinal flora [36,133]. It has been reported that fungal polysaccharides have prebiotic properties. They stimulate growth of *Lactobacillus* more strongly than the popular inulin and are also resistant to gastric juices (remaining more than 90% undigested) [36]. Nowak et al. studied polysaccharides isolated from 53 species of Polish mushrooms, and the results indicate that these compounds are a substrate for growth of the reference strain *L. acidophilus* and two strains of *L. rhamnosus* [36]. Synytsya et al. (2009) studied the prebiotic properties of various extracts of *P. ostreatus* and *P. eryngii* mushrooms, including alkali-soluble extracts containing (1→3)-α-d-glucans. The extracts were tested on nine probiotic strains of *Lactobacillus, Bifidobacterium*, and *Enterococcus*. In most cases, the fungal extracts stimulated the growth of probiotic bacteria, and the intensity of growth depended on the type of the extract used. In general, the extract from P. eryngii had better prebiotic properties. Studies show that (1→3)-α-d-glucans are prebiotic compounds and can stimulate the growth of *Lactobacillus* and *Bifidobacterium* bacteria [84].

### 6.4. (1→3)-α-d-Glucans as a Support for Enzyme Immobilization

The presence of free hydroxyl groups in the structure of α-glucans means they have the ability to form bonds with various molecules, and this property of polysaccharides is used for the immobilization of enzymes [134]. Immobilization is a common treatment to stabilize enzymes, and it is based on the high affinity between the ligand and biomolecule [135]. This process protects the enzymes against denaturation [136], which makes them cheaper and more stable during use and storage in comparison to the native enzyme [137]. Most fungi cell walls have α-glucans (especially *Laetiporus sulphureus*) [13], and they can be easy to obtain. For this reason, these polymers may be successfully used to support enzymes, and studies have confirmed this. Wang et al. (2006) report that α-glucan from *Penicillium chrysongenum* is highly effective for the immobilization of lipase from *Candida sp*. The results show increased stability during storage and improved thermal stability of immobilized lipase, although the enzyme immobilized at the same time had a slightly lower specific activity than the free enzyme (down 4.1%). According to the authors, these mobilized enzymes may be effectively used as a biocatalyst [134].

## 7. Sorption Properties of (1→3)-α-d-Glucans

The ability of fungi to bind metals has been known for a long time. Until now, research has focused on the content of metals in the whole fruiting bodies and has not considered a specific component responsible for sorption capacity [138,139,140]. It is now known that (1→3)-α-d-glucans have the ability to bind metals thus constituting a structural component responsible for the accumulation of elements in fungi [37,38]. In nature, there are two mechanisms by which fungi accumulate heavy metals: passive uptake (biosorption) and active uptake. In the former, heavy metals can be captured in the cellular structure and then biosorbed onto biding sites. In addition, this process is energy independent. The mechanisms of biosorption are generally based on physicochemical interactions between the metal ions and functional groups present on the cell surface, such as electrostatic interactions, ion exchange, and metal ion chelation or complexation [141]. Most literature defines the mechanism of metal uptake by viable cells as a two-phase process—an initial rapid stage of biosorption, followed by slower active uptake [142,143,144]. Humans have used the ability of fungi to bind metals through bioremediation, a process which uses biological material for purification of the environment, targeting various types of pollution (organic and inorganic pollution) [145,146,147]. By using fungal material as a sorbent, heavy metals can be removed (e.g., from industrial wastewater), and, at the same time, waste can be managed. In the sorption process, it is important to choose the sorbent and external conditions, such as pH, temperature and process time. Studies have been conducted on many (1→3)-α-d-glucans isolated from different species of fungi, and these indicate the most effective sorbent to be (1→3)-α-d-glucans from *Boletus edulis* and *Lentinus edodes* [37]. The sorption abilities of these polymers result from the developed and porous surface of glucan and a large number of functional groups on the surface, which contain oxygen. An interesting result of the research is the correlation between low crystallinity of studied polysaccharides and good sorption properties [37,38].

## 8. Conclusions

This review complements and systematizes existing knowledge of (1→3)-α-d-glucans, especially in terms of their application and function. (1→3)-α-glucans are glucose polymers with 1,3- glycosidic bonds, but 1,4- and 1,6-bonds can also be found in small amounts. Depending on the species of fungus, the molecular weight of α-glucan ranges from several to 850 kDa, as does the degree of polymerization, which ranges from 60 to 3500. Several methods of α-glucan isolation are reported in the literature, but these are generally based on separately water-soluble fractions and on preparation of alkaline-soluble (1→3)-α-glucans. (1→3)-α-glucans can be an inducer of (1→3)-α-glucanases, which can be used in the cleaning of teeth through decomposition of dental plaque mutans. Due to their structure, α-glucans free hydroxyl groups are capable of immobilizing enzymes, giving them more stability and reducing susceptibility to denaturation. These polymers potentiate immune responses and have prebiotic properties. However, α-glucans also have a negative effect. For example, they are involved in infection of plants by fungi and can be responsible for the pathogenicity of *Aspergillus fumigatus*. 

## Figures and Tables

**Table 1:** Review of fungal species that contain (1→3)-α-d-glucans, along with their brief characteristics.

Species of Fungus.	Molecular Mass Weight [kDa]	Viscosity [mPa⋅s]	Optical Rotation [α]${}_{D}^{25}$ [°]	(1→3)-α-d-glucan Content in Fungus (Dry Fungal Mass %)	Structure	Reference
*Aspergillus fumigatus*		16.7	+286	12.5	The backbone chain is formed mainly (91.3–97.8%) of glucose linked by (1→3), while (1→4) linkages are in the minority (1.3–7.2%). There are small amounts of three types of doubly substituted glucose residues, i.e., →2,3)-Glc*p*-(1→; →3,4)-Glc*p*-(1→ and →3,6)-Glc*p*-(1→.	[16]
*Aspergillus nidulans*		12.2	+384	9.4	as above	[16]
*Aspergillus niger*		8.8	+254	8.4	as above	[16]
*Aspergillus wentii*	850	17.0	+216	6.5	A linear polymer with 25 subunits; each subunit is constructed of about 200 residues of (1→3)- α-d-glucoses separated by a short spacer of (1→4)-α-d-glucoses.	[7]
*Cerrena unicolor*		2.12 (Fruiting body) 7.55 (Mycelium)	+206 (Fruiting body) +200 (Mycelium)	46.1	About 90% of the (1→3)- linkages; there are also →4)-α-d-Glc*p*-(1→ (7.4–4.4%) and →3,4)-α-d-Glc*p*-(1→(3.2–2.5%).	[18]
*Ganoderma lucidum*		1.94–1.98	+25 to +39	1.53–3.06	The backbone chain is formed mainly (74.9–87.9%) of glucose linked by (1→3)-, while (1→4)- linkages are in the minority (6.7–8.7%). There are small amounts of three types of doubly substituted glucose residues, i.e., →2,3)-Glc*p*-(1→; →3,4)-Glc*p*-(1→and 3,6)-Glc*p*-(1→.	[19]
*Lentinus edodes*	72.4–521			9	The chain consists mainly of (1→3)-bonds (67%) with a small number of (1→4)-bonds (27.3%) and →3,6)-Glc*p*-(1→ and →4,6)-Glc*p*-(1→.	[20,21,22]
*Laetiporus sulphureus*				57	The chain consists mainly of (1→3)-bonds (91.2%) and a small number of (1→4)-bonds (3%).	[22]
*Penicillium chrysogenum*	180			6		[23]
*Fomitopsis betulina* *(earlier Piptoporus betulinus)*	270			24	The chain consists mainly of (1→3)-bonds (84.6%) and a small number of (1→4)-bonds (6%).	[22,24]
*Pleurotus citrinopileatus*				4.0	91.2% of the (1→3)- linkages	[25]
*Pleurotus djamor*				3.1	73.8% of the (1→3)- linkages	[25]
*Pleurotus eryngii*				2.0	89.4% of the (1→3)- linkages	[25]
*Pleurotus ostreatus*				6.1	The chain consists mainly of (1→3)-bonds (82.8%) and a small number of (1→4)-bonds (7.4%).	[22]
*Pleurotus precoce*				2.7	84.7% of the (1→3)-linkages	[25]
*Ramaria botrytis*					A linear polymer is composed of →3)-α-d-Glc*p*-(1→ repeating units.	[17]

**Table 2:** Summary of α-glucans isolation methods detailing the reagents used in each isolation step.

Species of Fungi Used	Decolorization and Removal of the Water-Soluble Fraction	Neutralization Stage	Rinsing Stage	Reference
*Trichoderma viride*	Sodium borohydride, Sodium hydroxide, methanol, methanol water solution	Methanol-acetic acid solution	Methanol-water solution Water, boiled in water Ethanol	[39]
*Amanita muscaria*	Methanol, 0.9% sodium hydroxide, hot water, 5% Na_2_CO_3_ 1M NaOH solution with sodium borohydride (200 mg)	1M HCl	Water	[40]
*Schizophyllum commune*	Water 5% KOH activated charcoal	Acetic acid	Water	[41]
*Laetiporus sulphureus*	Water NaOH	HCl	Water	[42]

**Table 3:** Medical properties and potential application of fungal (1→3)-α-d-glucans.

Medicinal Property of (1→3)-α-d-Glucans	Application	Reference
Anti-tumour properties	Potential anti-cancer drug	[22,25,51,52,53,67,68,69,70,71,72]
Immunological activity	Adjuvants in vaccination	[16,25,54,67,68,69,70,71,72,73,74,75]
*Mutanase inducers*	Active ingredient of oral hygiene products (mouthwashes, toothpastes, or chewing gums),	[33,34,42,64,65,66,76,77,78]
Role in the pathogenicity of *Aspergillus fumigatus*	Vaccine and diagnostic test systems	[57,60,79,80,81,82,83]
Prebiotic properties	New prebiotic source	[36,84]
